# Design and fabrication of a magnetic nanobiocomposite based on flaxseed mucilage hydrogel and silk fibroin for biomedical and in-vitro hyperthermia applications

**DOI:** 10.1038/s41598-023-46445-w

**Published:** 2023-11-27

**Authors:** Fateme Radinekiyan, Reza Eivazzadeh-Keihan, Mohammad Reza Naimi-Jamal, Hooman Aghamirza Moghim Aliabadi, Milad Salimi Bani, Shirin Shojaei, Ali Maleki

**Affiliations:** 1 Research Laboratory of Green Organic Synthesis and Polymers, Department of Chemistry, Iran University of Science and Technology, P.O. Box 16846-13114, Tehran, Iran; 2 Catalysts and Organic Synthesis Research Laboratory, Department of Chemistry, Iran University of Science and Technology, Tehran, 16846-13114 Iran; 3 Advanced Chemical Studies Lab, Department of Chemistry, K. N. Toosi University of Technology, Tehran, Iran; 4 Department of Biomedical Engineering, Faculty of Engineering, University of Isfahan, Isfahan, Iran; 5 Medical School of Pharmacy, Nanotechnology Department, Kermanshah University of Medical Science, Kermanshah, Iran

**Keywords:** Biochemistry, Biological techniques, Chemistry, Nanoscience and technology

## Abstract

In this research work, a magnetic nanobiocomposite is designed and presented based on the extraction of flaxseed mucilage hydrogel, silk fibroin (SF), and Fe_3_O_4_ magnetic nanoparticles (Fe_3_O_4_ MNPs). The physiochemical features of magnetic flaxseed mucilage hydrogel/SF nanobiocomposite are evaluated by FT-IR, EDX, FE-SEM, TEM, XRD, VSM, and TG technical analyses. In addition to chemical characterization, given its natural-based composition, the in-vitro cytotoxicity and hemolysis assays are studied and the results are considerable. Following the use of highest concentration of magnetic flaxseed mucilage hydrogel/SF nanobiocomposite (1.75 mg/mL) and the cell viability percentage of two different cell lines including normal HEK293T cells (95.73%, 96.19%) and breast cancer BT549 cells (87.32%, 86.9%) in 2 and 3 days, it can be inferred that this magnetic nanobiocomposite is biocompatible with HEK293T cells and can inhibit the growth of BT549 cell lines. Besides, observing less than 5% of hemolytic effect can confirm its hemocompatibility. Furthermore, the high specific absorption rate value (107.8 W/g) at 200 kHz is generated by a determined concentration of this nanobiocomposite (1 mg/mL). According to these biological assays, this magnetic responsive cytocompatible composite can be contemplated as a high-potent substrate for further biomedical applications like magnetic hyperthermia treatment and tissue engineering.

## Introduction

**Figure 1:**
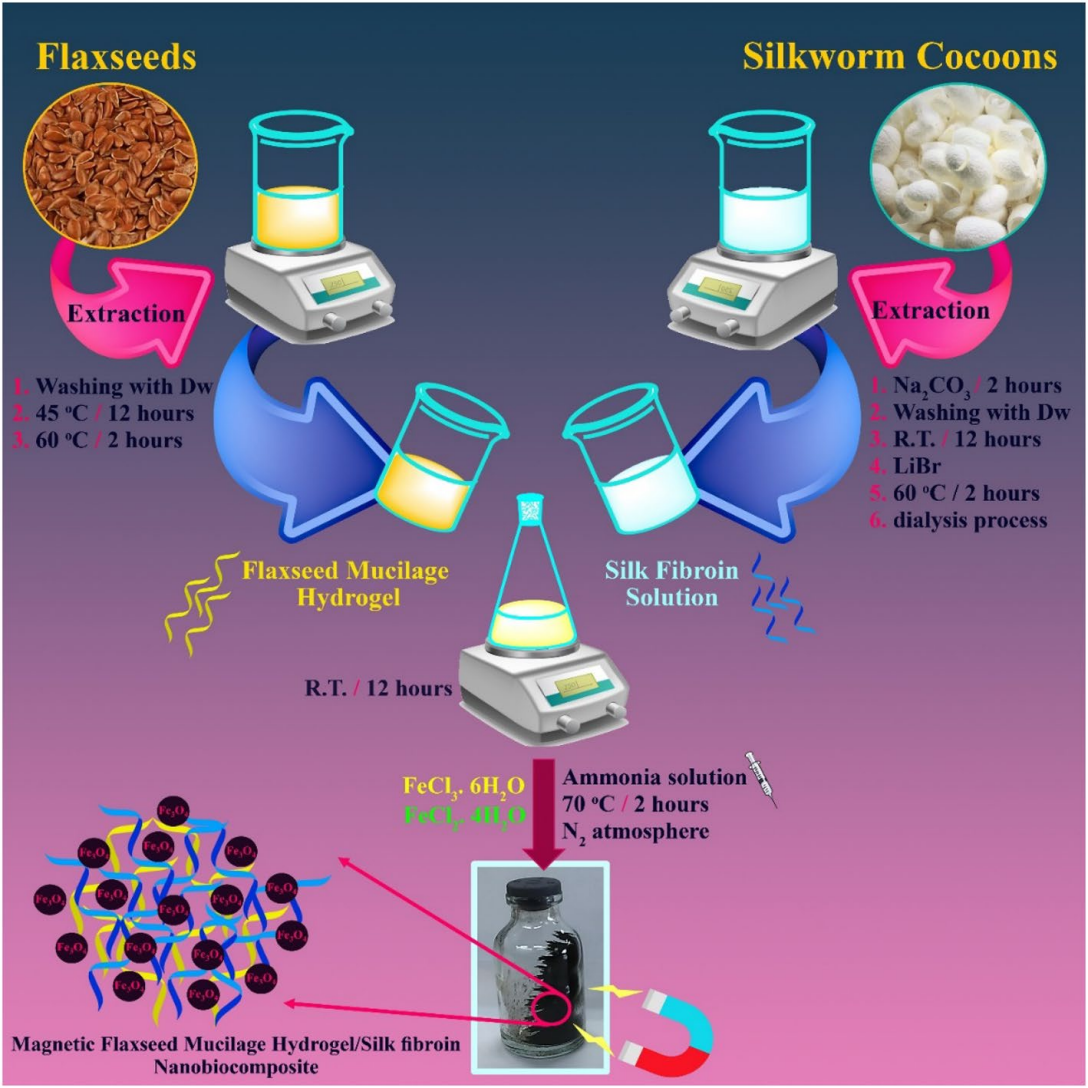
Synthesis process of magnetic flaxseed mucilage hydrogel/SF nanobiocomposite.

Since prehistoric times, herbal plants as natural origin sources have been a longstanding of interest due to their high potential capacity in extraction and developing a diversity of drugs and other chemical entities^1^. In other words, extracting and isolating various molecules and compounds such as plant polyphenols with high biological performances and the generation of new therapeutics have promoted the medical science, particularly in preventing and treating chronic diseases such as cancer^1–3^. In this regards, plant-derived mucilage as a rich source of polysaccharide hydrocolloids with jelly-like structure, can be extracted from the vegetative part of plants during the hydration process^4^. Mucilages contain large molecules mainly carbohydrates combined with uronic acids, glycoproteins, and important bioactive compounds^4^. Given their biodegradability, biocompatibility, non-toxicity, high water swelling ability, and mucoadhesive properties, these natural compounds have been applied extensively in food, cosmetics^4,5^, and pharmaceutical science^4,6^. Apart from their privileged functional properties, these natural compounds perform an impressive role in the formation of gel, emulsion, film, and coated metal nanoparticles due to their hydrogen bonding interactions between functional polar groups^7^. In comparison to other plant-based biopolymers, it has proven that to formulate new three-dimensional hydrogel networks, mucilages can act as a primary biopolymer or cross-linking agents because of its excellent biomedical activity and high stability^7^. Among this category, one of the oldest crops which is cultivated as a source of fiber, oil, and medicinal compounds, is dicotyledonous flax (*Linum usitatissimum *L.) plant. According to its remarkable biological activities, the entity of polysaccharides, cyclic peptides, cyanogenic glycosides, linolenic acid, alkaloids, and phenolic antioxidant compounds, its applicability has been extended in reducing cardiovascular diseases, cancer treatments, and supporting the immune systems^8,9^. Preclinical studies have stated that flaxseed can inhibit the growth of human breast cancer^10^, prostate carcinoma^11^, and metastasis of melanoma cells^12^. The polysaccharide part of flaxseed consists of soluble and insoluble fibers. Cellulose and lignin are included as insoluble fiber fraction; and the flaxseed mucilage hydrogel is known as a soluble fiber fraction containing two heterogenous polysaccharides [a neutral arabinoxylan (75%) and an acidic rhamnogalacturonan (25%)]^8,13,14^. These components have shown anti-tumor, anti-aging, and anti-oxidant activities and they can protect the liver^15^. Furthermore, among bioactive molecules in flaxseed mucilage, lignans as phenolic compounds contain secoisolaricirezinol (SECO) and the diglucoside secoisolaricirezinol (SDG), which are very strong antioxidants and phyto-estrogens. SECO, SDG, and gerbacin (8-hydroxycaempferol) as natural plant flavonoids can reduce blood cholesterol, protect cardiovascular system, aid kidney function and decrease the tumors growth of breast cancer^15^. Aside from natural polymeric plants with multifunctionality^16^, silk fibroin (SF) as a natural proteinous biomaterial with the classified structural conformation [silk I (random coil and α-helix) and silk II (β-sheet)], has been highlighted due to tunable biodegradability, excellent biocompatibility, stable mechanical property, and also preserving the biomolecular systems and their interactions^17^. Given the non-cytotoxicity, non-carcinogenicity, and low hemostatic features of this natural polymer, increasing the immunological response, adhesion, and cellular growth can be improved using SF^18–20^. Considering these descriptions, its ease of extraction, and modification, the USA Food and Drug Administration has approved this natural protein for suture materials and clinical trial assays^17^. The incorporation of SF with other materials such as graphene derivatives^21^, polymers^22,23^, and nanoparticles^24^ can ameliorate its antimicrobial activity and develop new SF-based nanocomposites. On the other hand, a variety of structural forms of this biopolymer including gels, films, microsphere, and scaffolds have been well-investigated in drug delivery, tissue engineering, and other biomedical applications^17,25^. As developed in the last decades, the hyperthermia treatment has been emerged as a complementary modality to treat advanced cancer diseases and enhance the quality of a patient's life^26,27^. Three categorized hyperthermia methods including local, regional, and whole-body hyperthermia can be employed as an effective therapeutic strategy alongside surgery, radiotherapy, and chemotherapy^27^. Hyper thermic therapy entails heating tumor cells to 40–45 °C in order to eliminate them via a sequence of thermally induced metabolic processes such as apoptosis^28,29^. All of things happen because the hyperthermia tries to change the extracellular microenvironment by triggering immune responses and causing tumor cells to switch to a metabolic system that doesn't use oxygen^30^. On the other side, local heating the cancer tissue by heating probes can damage to the healthy and surrounding tissue; therefore, the fabrication of new magnetic nano systems is required to target the cancerous tissue and can be controlled from outside of the body^31^. Recently, magnetic hyperthermia and the administration of Fe_3_O_4_ magnetic nanoparticles (Fe_3_O_4_ MNPs) as a heating mediator with tunable magnetic properties have been developed as a new therapeutic technique^32–34^. Different fundamental factors such as the specific absorption rate (SAR), alternating magnetic field (AMF), the concentration of these appealing magnetic candidates, and as well, the physiological features of medium can influence on their efficiency^33^. In this non-invasive treatment, by applying an AMF and oscillating the magnetic moment of these forefront nanoparticles, the magnetic energy converts to heating energy. The temperature of tumor area is locally raised to destroy the cancerous cells and preventing the collateral damage to healthy cells^35,36^. The maintenance of colloidal stability, biocompatibility, and assessment of possible cytotoxicity of MNPs in physiological conditions play a critical role in determining their suitability for in vivo applications. Within this particular context, several articles have concentrated on exploring potential techniques to enhance the physicochemical and biological characteristics of magnetic nanoparticles (MNPs) through the application of coatings^37^. Natural fibers, such as silk and linen, provide desirable attributes that make them well-suited for coating applications. These attributes include their exceptional biocompatibility as well as their abundant and readily available nature^38^. SF has remarkable mechanical qualities and stability, as well as possessing desirable attributes such as biocompatibility, biodegradability, and the ability to elicit minimal inflammatory reactions from the host organism. The notable benefits associated with silk fibroin have resulted in its consideration for a wide range of biological applications^39^. Flax mostly consists of polysaccharides, namely cellulose (about 64%) and hemicellulose (approximately 12%). These constituents contribute to its cost-effectiveness and provide exceptional physical–mechanical characteristics^40^. The use of a coated MNP enhances its targeting capabilities, therefore concomitantly augmenting the effectiveness of the therapy and mitigating its deleterious impact on neighboring healthy tissues^41^. It is conceivable that the formation of magnetic nanobiocomposite based on natural mucilages and SF polymer can be attended to synergistic outcomes in cancer treatment. In this research study, considering an enhanced deployment of flaxseed mucilage hydrogel as a valuable health-benefiting biomaterial, the excellent biological performance of SF biopolymer, and magnetic properties of Fe_3_O_4_ MNPs, magnetic flaxseed mucilage hydrogel/SF nanobiocomposite was designed and synthesized. Following the extraction of flaxseed mucilage hydrogel and SF solution, their composition was magnetized by in-situ formation of Fe_3_O_4_ MNPs (Fig. 1). Apart from characterizing the chemical structure (FT-IR, EDX, FE-SEM, TEM, XRD, VSM, and TG), different concentrations of designed magnetic nanobiocomposite showed eye-catching biological activities in the presence of two normal HEK293T cell and breast cancer BT549 cell lines. Observing considerable cell viability percentage for normal cells and inhibiting the growth of cancer cells confirmed its biocompatibility. As well as, the less hemolysis percentage disclosed that this magnetic nanobiocomposite could be considered as a hemocompatible nanostructure with red blood cells (RBCs). Moreover, using 1 mg/mL of magnetic flaxseed mucilage hydrogel/SF nanobiocomposite and generating a high SAR value (107.8 W/g), could disclose its capability in magnetic hyperthermia treatment.

## Result and discussion

### Characterization of magnetic flaxseed mucilage hydrogel/SF nanobiocomposite

By synthesizing the magnetic flaxseed mucilage hydrogel/SF nanobiocomposite in four synthesis steps (Fig. 1), the structural evaluation of this new magnetic nanobiocomposite such as the formation of new bonds and assigning new functional groups, elemental composition, characterizing the morphology, indexing crystalline peaks, magnetic properties, and thermogravimetric behaviour is conducted using FT-IR, EDX, FE-SEM, TEM, XRD, VSM, and TG analyses. The results are discussed as follows.

#### FT-IR analysis

**Figure 2:**
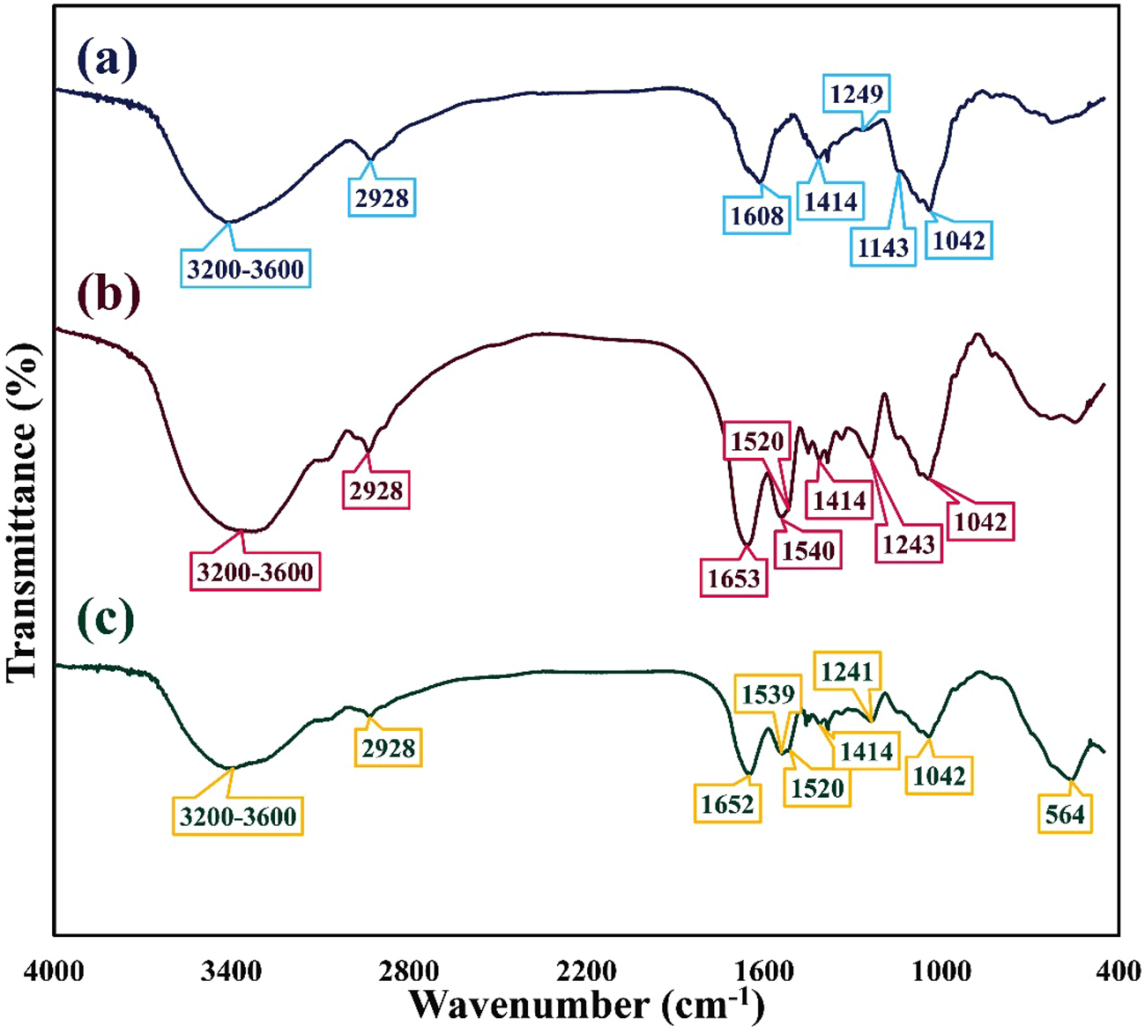
FT-IR spectra of (a) freeze-dried form of flaxseed mucilage hydrogel, (b) freeze-dried form of flaxseed mucilage hydrogel/SF structure, and (c) magnetic flaxseed mucilage hydrogel/SF nanobiocomposite.

The formation of new functional groups and monitoring the synthesis process of magnetic flaxseed mucilage hydrogel/SF nanobiocomposite, FT-IR spectra were recorded from each synthesis step. Figure 2a shows the FT-IR spectrum of freeze-dried form of flaxseed mucilage hydrogel. As can be seen, a broad band at region of 3200–3600 cm^−1^ and a small band at 2928 cm^−1^ are related to the stretching vibration mode of O–H groups of aliphatic alcohol and symmetrical stretching vibration mode of the C–H bond of CH_2_-CH_3_ groups in aliphatic chains^42^. An absorption band at 1414 cm^−1^ is attributed to the scissor-type vibration mode of CH_2_ groups^42^. C=O elongation vibration mode of galacturonic acid groups and the contribution of angular deformation of water molecules, symmetric and asymmetric stretching vibration modes of C–O–C glycosidic bonds are confirmed by observing an intense absorption band at 1608 cm^−1^, a small and strong absorption bands at 1249 cm^−1^ and 1042 cm^−1^^42,43^. Additionally, a small absorption band at 1143 cm^−1^ is assigned to the vibration mode of C–O–C glycosidic linkage bond^42,43^. The composition of flaxseed mucilage hydrogel and extracted SF was accompanied by the formation of new bands (Fig. 2b). Normally, three vibrational bands in different regions of FT-IR spectrum can specify the structural conformation of extracted SF^44,45^. The C–N stretching vibration mode of amide III (1230–1270 cm^−1^), the N–H bending vibration mode of amide II (1520–1540 cm^−1^), and the C=O stretching vibration mode of amide I (1625–1660 cm^−1^) are considered as these three main vibrational bands^44,45^. As illustrated in Fig. 2b, apart from the absorption bands of flaxseed mucilage hydrogel (1042 cm^−1^, 1414 cm^−1^, 2928 cm^−1^), the intense C=O stretching vibration mode of amide I at 1653 cm^−1^ which has covered C=O elongation vibration mode of galacturonic acid groups, and the C–N stretching vibration mode of amide III at 1243 cm^−1^ confirm the presence of random coil and α-helix conformations respectively^45^. Also, the two N–H bending vibration modes of amide II at 1520 cm^−1^ and 1540 cm^−1^ are sequentially ascribed to the β-sheet and random coil conformations of SF structure^45^. On the other side, the broad absorption band around 3200 cm^−1^ to 3600 cm^−1^ related to the hydroxyl groups, affirms the presence of β-sheet conformation^46^. Besides, it can be mentioned that increasing the intensity of O–H absorption band can be related to the formation of hydrogen bonds and other intermolecular interactions between flaxseed mucilage hydrogel and SF structure^46^. Aside from the mentioned absorption bands, by magnetizing the flaxseed mucilage hydrogel/SF composition, a sharp absorption band at 564 cm^−1^ which is assigned as Fe–O stretching vibration mode, confirms the presence of Fe_3_O_4_ MNPs in the structure of magnetic nanobiocomposite (Fig. 2c)^47^.

#### EDX analysis

**Figure 3:**
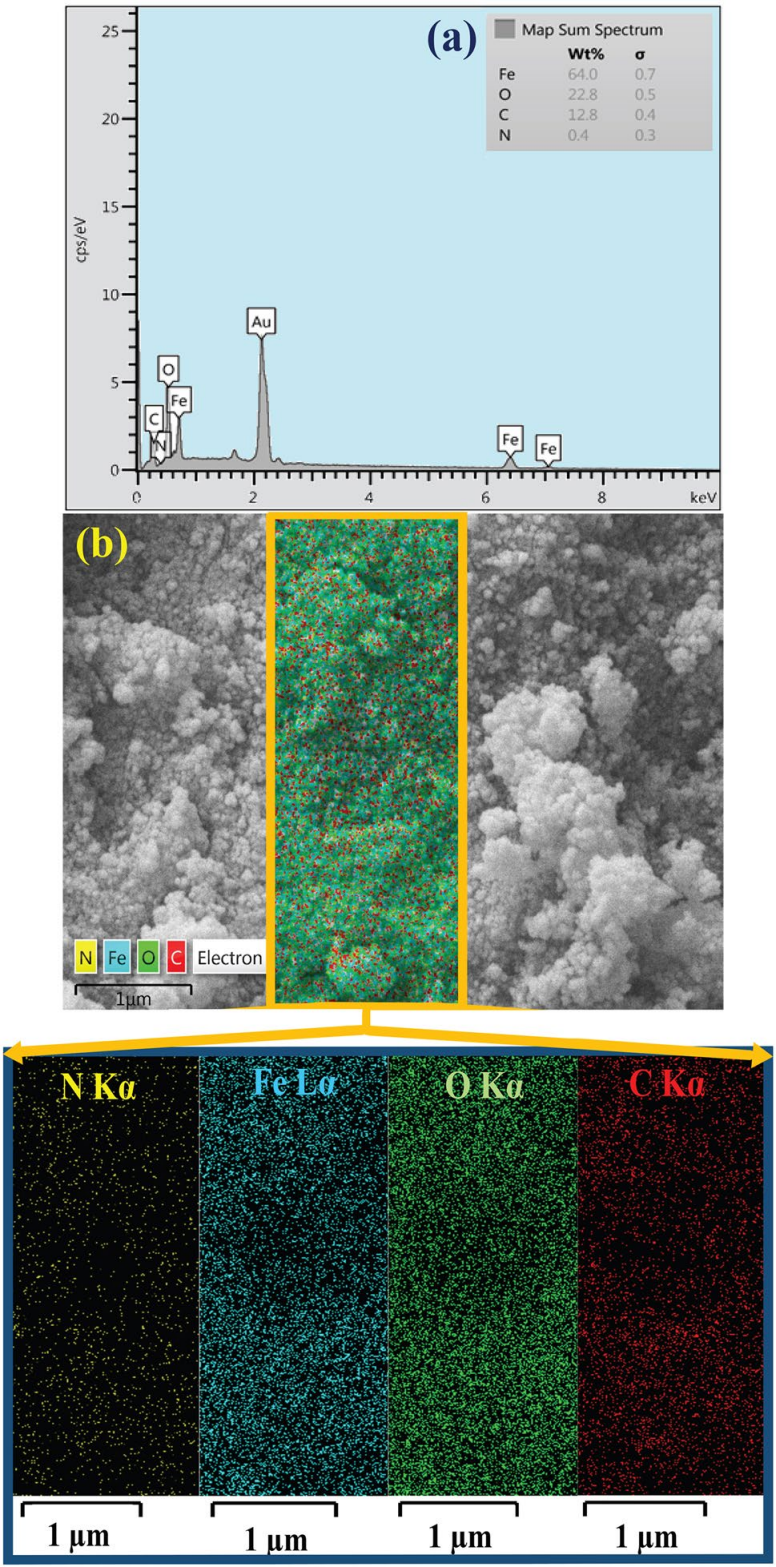
**(a**) EDX spectrum and (**b**) elemental mapping images of magnetic flaxseed mucilage hydrogel/SF nanobiocomposite.

The structural elements of magnetic flaxseed mucilage hydrogel/SF nanobiocomposite and the weight percentage of identified elements were qualitatively studied by EDX analysis (Fig. 3a,b). As determined in EDX spectrum (Fig. 3a), assigning carbon and oxygen peaks can be attributed to the presence of extracted flaxseed mucilage and natural SF polymer in the structure of nanobiocomposite. Moreover, according to the functional amino acid sequences in the SF structure, the nitrogen peak can affirm this natural biopolymer. Furthermore, observing two iron peaks and also oxygen peak is related to the synthesized Fe_3_O_4_ MNPs embedded in the structure of nanobiocomposite. Apart from EDX spectrum, elemental mapping images shows well the distribution pattern of assigned elements (Fig. 3b).

#### FE-SEM and TEM imaging

**Figure 4:**
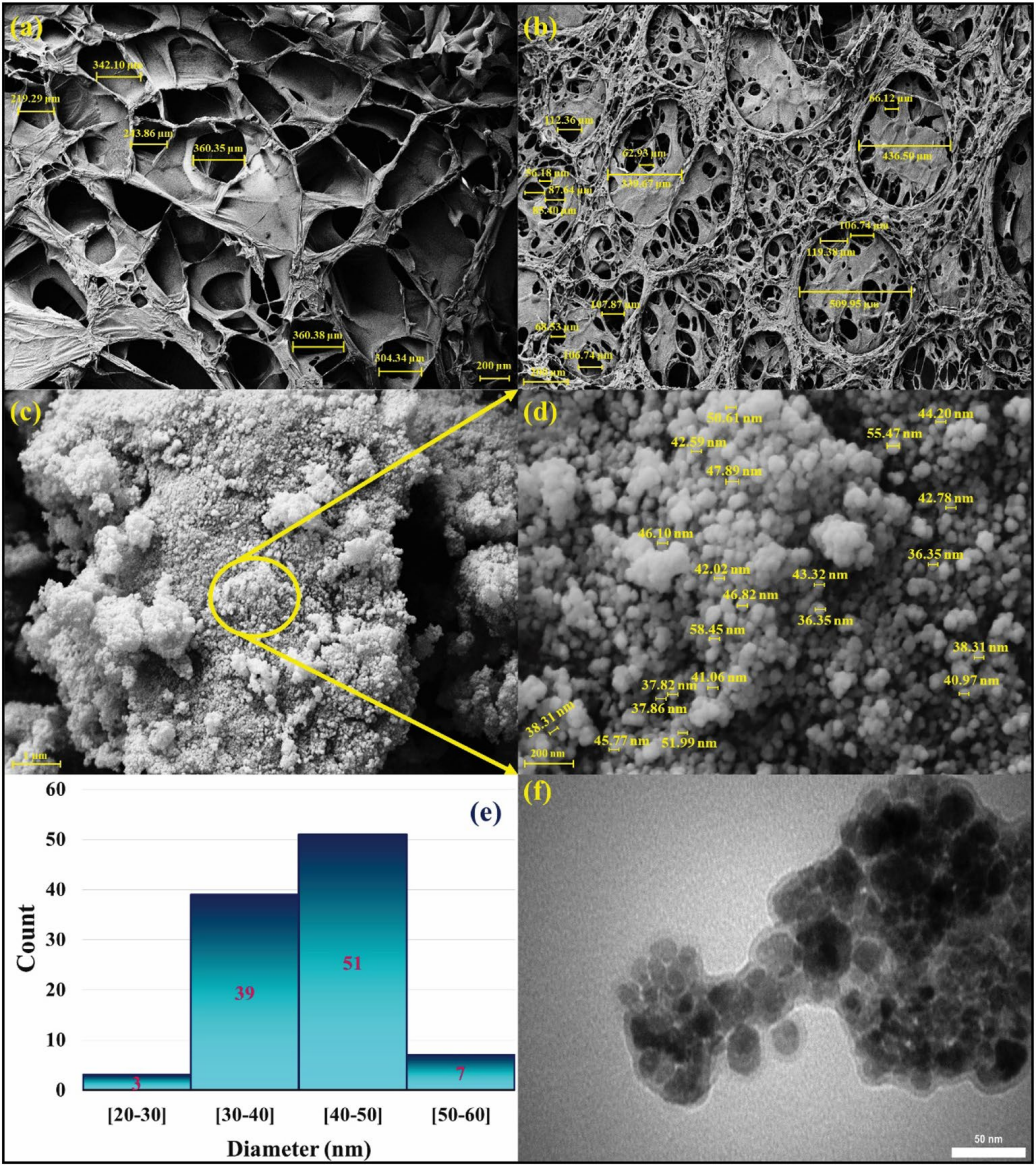
FE-SEM images of freezed-dried forms of **(a**) flaxseed mucilage hydrogel, (**b**) flaxseed mucilage hydrogel/SF structure, (**c**,**d**) magnetic flaxseed mucilage hydrogel/SF nanobiocomposite, (**e**) size distribution histogram chart of magnetic flaxseed mucilage hydrogel/SF nanobiocomposite, and (**f**) TEM image of magnetic flaxseed mucilage hydrogel/SF nanobiocomposite.

To evaluate the morphology and monitor the structure, FE-SEM imaging was taken from each synthesis step (Fig. 4a–d). As determined in the Fig. 4a, the channel-like morphology can be seen for the freezed-dried form of flaxseed mucilage hydrogel. By combining and modification of flaxseed mucilage hydrogel with extracted SF solution, the morphology, multiplicity and variety of pores have changed at the same scale (200 µm) (Fig. 4b). It is true that different size of pores (smaller or bigger than pores of flaxseed mucilage hydrogel) is observed by adding silk fibroin solution (Fig. 4a,b); however, it can be seen that the bigger pores of flaxseed have been filled with lots of silk fibroin chains resulting in formation of smaller pores. Given these observations, it can be inferred that the presence of polymeric chains of silk fibroin and formation of more hydrogen bonding interaction with flaxseed mucilage polymers has caused to bring these polymeric chains closer and therefore, the size of the pores has become smaller and the porosity has increased. This composition substrate with web-like morphology and extended porosity can supply an appropriate medium with biological sustainability for the regulation of adhesion and cellular proliferation (Fig. 4b)^48^. Also, given the previous research studies, it is reported that the degradation process of silk fibroin-based substrates with smaller pores is slower than those with bigger pores. Controlling the degradation rate of a biomaterial substrate is a crucial factor; that must be considered for advanced biomedical applications such as tissue engineering^49^. Following the in-situ formation of Fe_3_O_4_ MNPs in the presence of natural composite substrate and the formation of magnetic flaxseed mucilage hydrogel/SF nanobiocomposite, as can be observed in Fig. 4c,d, an almost spherical and uniform morphology have covered the pores and pore interconnectivity. Furthermore, the average size of prepared magnetic nanobiocomposite has estimated between 40 to 50 nm as specified by the size distribution histogram chart (Fig. 4e). In continuation, TEM image of magnetic flaxseed mucilage hydrogel/SF nanobiocomposite (Fig. 4f) was assessed according to the described FE-SEM images (Fig. 4c,d). In this connection, Fe_3_O_4_ MNPs with almost uniform distribution are observed in the structure of flaxseed mucilage hydrogel/SF substrate. Moreover, in line with this observation, it can be noted that flaxseed mucilage hydrogel/SF substrate has placed these almost uniform nanoparticles in itself; in such a way that Fe_3_O_4_ MNPs with the core–shell structure can be seen (Fig. 4f).

#### XRD pattern

**Figure 5:**
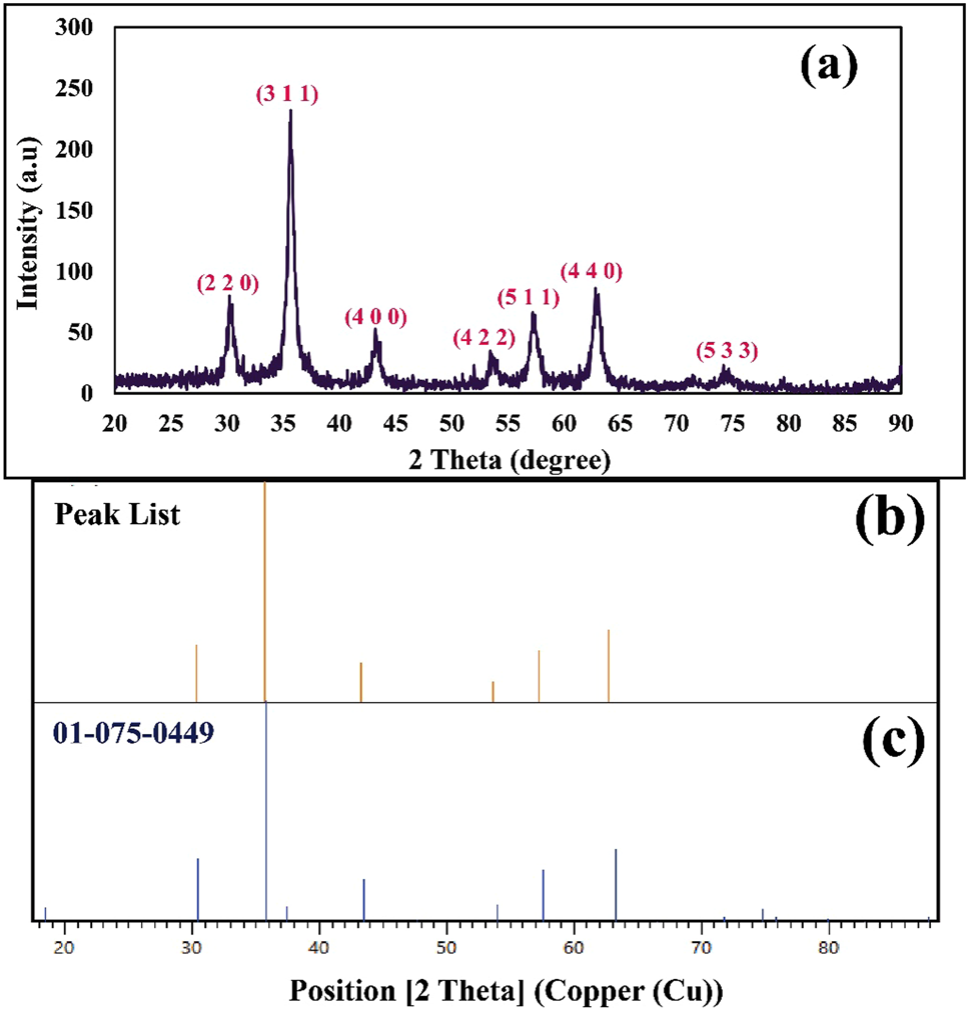
(**a**) XRD pattern of magnetic flaxseed mucilage hydrogel/SF nanobiocomposite, (**b**) peak list of magnetic flaxseed mucilage hydrogel/SF nanobiocomposite, and (**c**) the reference of Fe_3_O_4_ MNPs in the structure of magnetic flaxseed mucilage hydrogel/SF nanobiocomposite.

XRD pattern of magnetic flaxseed mucilage hydrogel/SF nanobiocomposite was analysed to evaluate its crystalline phase and structure (Fig. 5a–c). As illustrated in Fig. 5a, the crystalline identified peaks at determined diffraction peaks at 2Ө = 30.21°, 35.62°, 43.26°, 53.62°, 53.70°, 57.37°, 62.90°, and 74.28° are complied perfectly with the standard pattern of Fe_3_O_4_ MNPs (JCPDS card No. 01-075-0449) (Fig. 5b,c)^50^. In parallel, the Miller indices including (2 2 0), (3 1 1), (4 0 0), (4 2 2), (5 1 1), (4 4 0), and (5 3 3) are assigned for each identified peak (Fig. 5a).

#### VSM and thermogravimetric analyses

**Figure 6:**
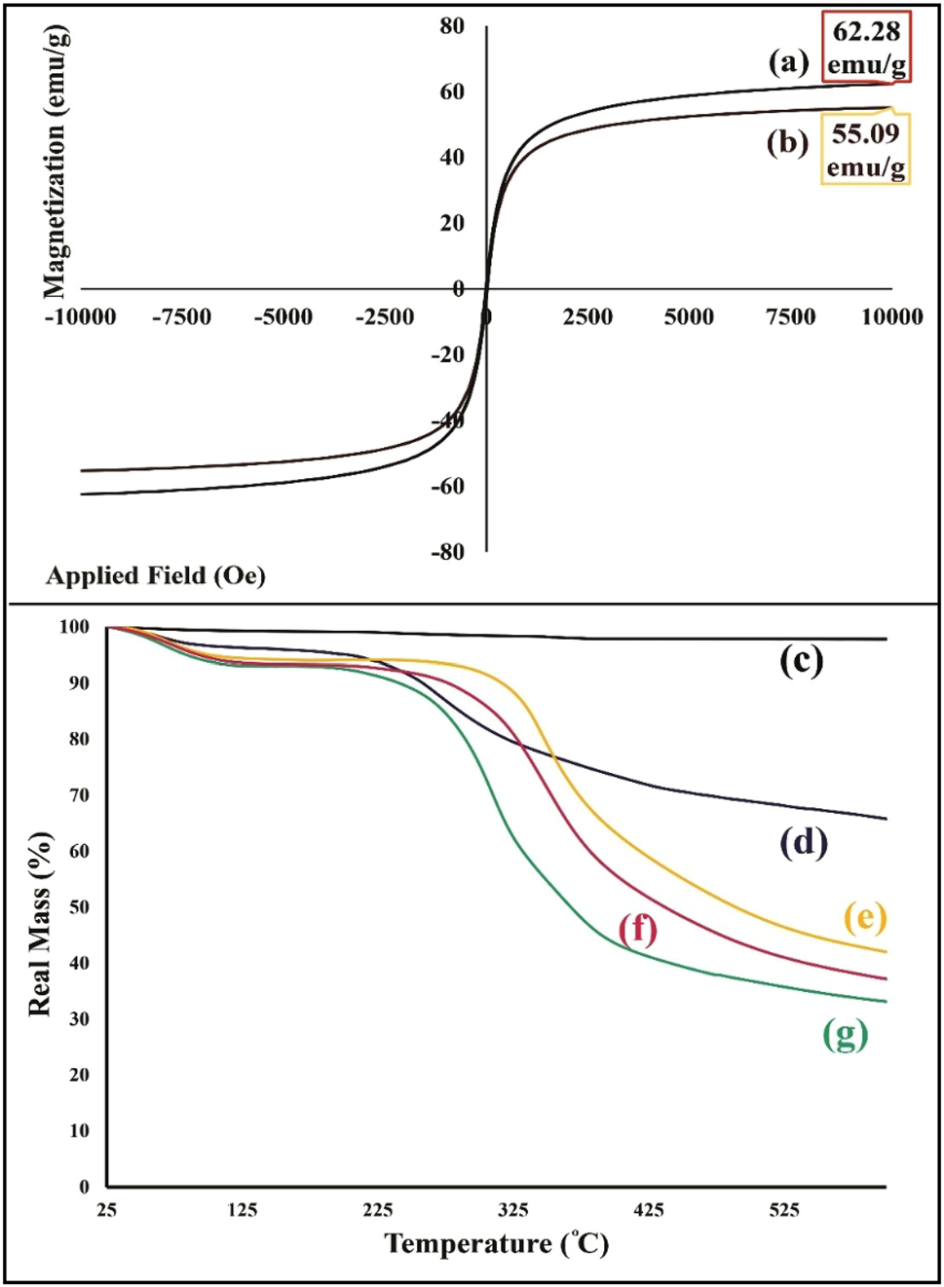
Hysteresis loop curves of (a) bare Fe_3_O_4_ magnetic nanoparticles, and (b) magnetic flaxseed mucilage hydrogel/SF nanobiocomposite; thermogravimetric curves of (c) bare Fe_3_O_4_ MNPs, (d) magnetic flaxseed mucilage hydrogel/SF nanobiocomposite, (e) freeze-dried form of silk fibroin solution, (f) freeze-dried form of flaxseed mucilage hydrogel/SF substrate, and (g) freeze-dried form of flaxseed mucilage hydrogel.

Normally, dynamic magnetic features of nanoparticles can be impacted by various essential factors such as a core size, an interparticle distance, a shell thickness, the crystalline structure of iron groups, and the interparticle and interparticle interactions^51^. The saturation magnetization values of bare Fe_3_O_4_ MNPs and magnetic flaxseed mucilage hydrogel/SF nanobiocomposite as an important magnetic parameter was measured by VSM instrument and applying a magnetic field between -10 kOe to + 10 kOe (Fig. 6a,b). According to their hysteresis loop curves and compared to the saturation magnetization value of bare Fe_3_O_4_ MNPs (62.28 emu/g), the saturation magnetization value of magnetic flaxseed mucilage hydrogel/SF nanobiocomposite (55.09 emu/g) has decreased. It can be deduced that this reduction can be attributed to the in-situ synthesis of Fe_3_O_4_ MNPs in the presence of flaxseed mucilage hydrogel/SF structure and covering Fe_3_O_4_ MNPs by a neat shell of this substrate composition. Figure 6c–g shows the thermogravimetric behaviour of bare Fe_3_O_4_ MNPs, magnetic flaxseed mucilage hydrogel/SF nanobiocomposite, freeze-dried forms of SF solution, flaxseed mucilage hydrogel/SF substrate, and flaxseed mucilage hydrogel. In Fig. 6c, no specific mass reduction is observed for Fe_3_O_4_ MNPs at temperature range of 25 °C to 600 °C and these nanoparticles are stable. On the other side, the thermogravimetric curves of magnetic flaxseed mucilage hydrogel/SF nanobiocomposite, freeze-dried form of flaxseed mucilage hydrogel/SF, and its polymeric components start with same behaviour (Fig. 6d–g). the first mass reduction less than 5% at temperature of 25 °C to almost 125 °C can be related to the evaporation of absorbed water molecules^52^. The second mass reduction (almost 30%) at temperature range of 200 °C to almost 500 °C, is corresponded to the degradation of saccharide rings, the disintegration of polymeric chains of flaxseed mucilage extract, and as well, cleaving the peptide bonds of SF, and breaking down the side chain sequences of amino acid presented in the structure of magnetic flaxseed mucilage hydrogel/SF nanobiocomposite (Fig. 6d)^53,54^. The remaining mass (almost 65%) can be related to the Fe_3_O_4_ MNPs presented in the structure of designed nanobiocomposite. Given the mentioned mass reduction and remaining mass percentages, it can be deduced that there is a conformity between the results of TG and EDX analyses. However, the second mass reduction in flaxseed mucilage hydrogel, flaxseed mucilage hydrogel/SF substrate, and SF is almost 50%. Moreover, compared to flaxseed mucilage hydrogel, this mass reduction in the thermogravimetric curves of flaxseed mucilage hydrogel/SF substrate, and SF is started at higher temperature (Fig. 6e–g).

### Bio-application of magnetic flaxseed mucilage hydrogel/SF nanobiocomposite

#### In-vitro cytotoxicity assay results

**Figure 7:**
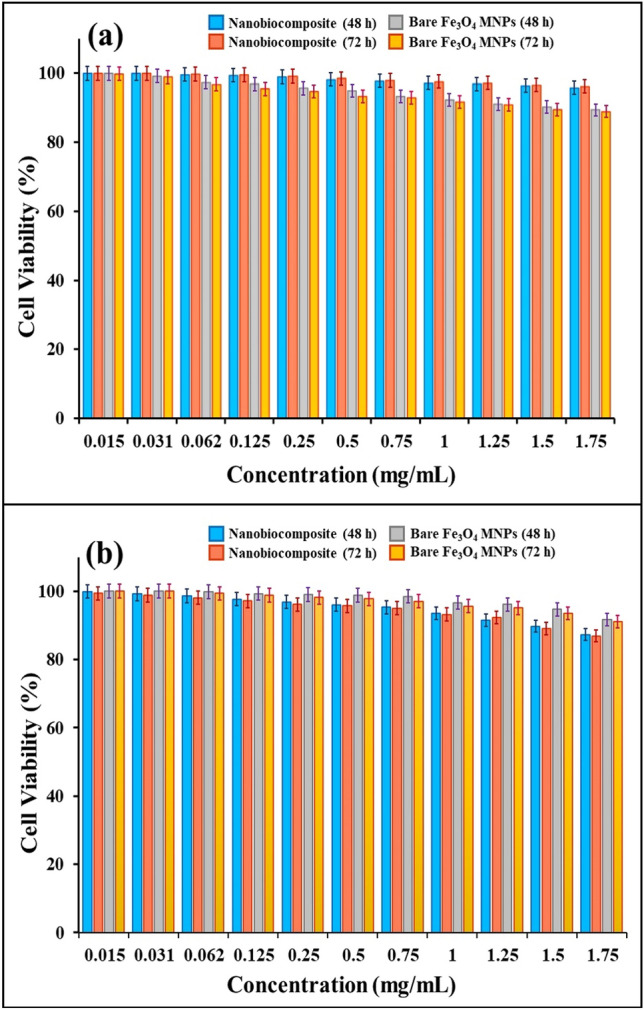
Cell viability percentages of (**a**) HEK293T cells, (**b**) BT549 cells after treatment with magnetic flaxseed mucilage hydrogel/SF nanobiocomposite and bare Fe_3_O_4_ MNPs in 2 and 3 days (very significant compared to positive control, *P* ≤ 0.001) at different concentrations.

The cytotoxicity assay results from bare Fe_3_O_4_ MNPs and magnetic flaxseed hydrogel/SF nanobiocomposite show that, the cell viability percentage of HEK293T cells treated with 1.75 mg/mL of this nanobiocomposite, after 2 and 3 days is 95.73 ± 0.16% and 96.19 ± 0.14% (insignificant compared to negative control, *P* ≥ 0.05); whereas for the BT549 cells, these values are 87.32 ± 0.29% and 86.9 ± 0.26% (significant compared to negative control, *P* ≤ 0.05) respectively (Fig. 7a,b). However, the toxicity of bare Fe_3_O_4_ MNPs against normal HEK293T cell lines is higher and they cannot reduce the survival rate of cancer BT549 cells compared to magnetic flaxseed hydrogel/SF nanobiocomposite. Therefore, it can be deduced that this synthesized nanobiocomposite has more biocompatibility with HEK293T cell line and also, this nanostructure is able to inhibit the growth of BT549 cells and decrease their survival rate.

#### In-vitro hemocompatibility assays results

**Figure 8:**
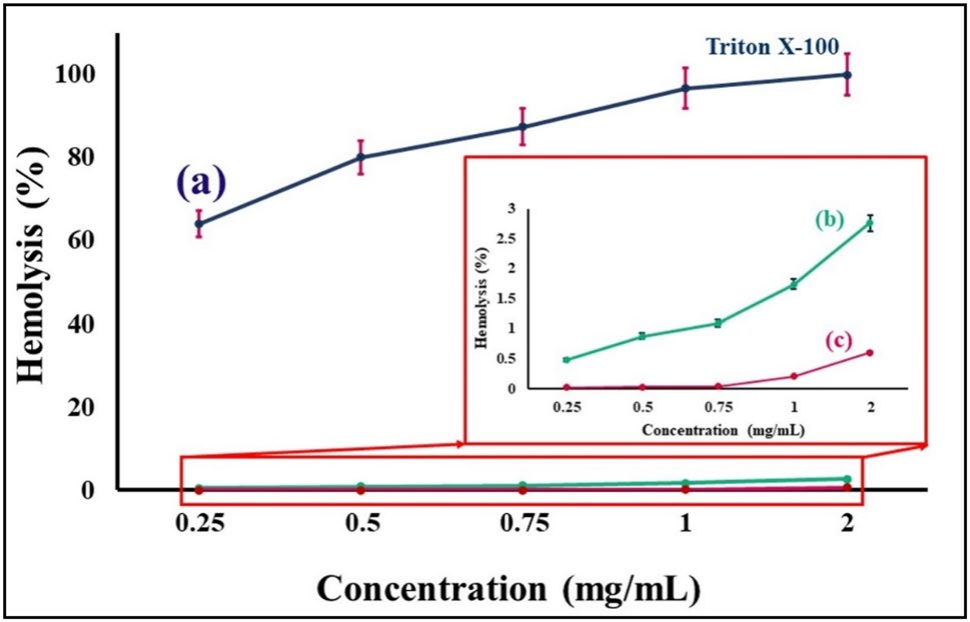
Hemolysis percentage graphs of (a) Triton X-100 (positive control), (b) bare Fe_3_O_4_ MNPs, and (c) magnetic flaxseed hydrogel/SF nanobiocomposite at different concentrations.

The hemolysis study of bare Fe_3_O_4_ MNPs and magnetic flaxseed mucilage hydrogel/SF nanobiocomposite was conducted to evaluate their hemocompatibility with RBCs. The results are the mean of three independent experiments. As can be seen in Fig. 8a–c, Triton X-100 (as a positive control) lyses almost all RBCs (Fig. 8a); whereas, magnetic flaxseed mucilage hydrogel/SF nanocomposite shows almost no hemolytic effect even at the highest concentration (2 mg/mL) (Fig. 8b,c). Furthermore, in comparison to magnetic flaxseed mucilage hydrogel/SF nanobiocomposite, it can be understood that the hemolytic activity of Fe_3_O_4_ MNPs is more than 4 times. According to the ISO standard, document 10 993–5 1992^55^, if the hemolysis rate of a substance is less than 5%, it is considered safe and hemocompatible. Although, it is true that 2 mg/mL of Fe_3_O_4_ MNPs is safe, however, the compatibility of magnetic flaxseed mucilage hydrogel/SF nanobiocomposite with erythrocyte cell is more. As results of these observations, it can be concluded that the presence of flaxseed mucilage hydrogel/SF substrate can reduce the hemolysis percentage of this magnetic nanobiocomposite and improve its hemocompatibility.

#### Heating capacity under the AMF

Magnetic hyperthermia is caused by three processes, Néel relaxation, Brownian relaxation, and hysteresis loss. The size, shape, crystallographic anisotropy of nanoparticles, and their degree of aggregation or agglomeration, all have a significant impact on how much these nanoparticles contribute^56^. It might be able to find a size threshold for a nanoparticle above which the loss of hysteresis has a big effect. Below this critical limit, relaxation mechanisms would take over that make the magnetic moment spin when it is in a magnetic field. If the magnetization rotates while the particle remains stationary, Neel Relaxation will allow heat to escape. Otherwise, the particle spins and the magnetic moment remains constant in reference to the crystallographic axes via which Brownian relaxation occurs. The specific absorption ratio (SAR), which is the rate of heat production, is used to figure out how well magnetic nanoparticles heat. The specific absorption rate (SAR) was calculated using Eq. (1).

1 $$\mathrm{SAR}=\frac{C}{m}\frac{\Delta T}{\Delta t}$$

where C (4.185 J/(g °C)) is the specific heating capacity of the fluid, m is the concentration of magnetic nanoparticles and ∆T is the temperature change in the time span ∆t.

In this research study, different experiments are carried out to evaluate the heating capacity of magnetic flaxseed hydrogel/SF nanobiocomposite. An oscillating magnetic field is applied to numerous samples with concentrations of 1 mg/mL (m) and a surrounding fluid temperature (23 °C) at the start. Different field frequencies (100 kHz, 200 kHz, 300 kHz, and 400 kHz) are employed with constant field intensities to see how they affect variables. During the ten minutes of exposure, the temperature of the fluid around the item is checked every five minutes, giving us two different time periods. As can be observed in the supplementary information file, Fig. S1a, when the magnetic field is introduced, the temperature increases significantly. According to the initial temperature (23 °C), at 100 kHz, which is 4.65 °C, the temperature considerably goes up at the first time period. For 200 kHz, 300 kHz, and 400 kHz, the values are 1.16 °C, 1.36 °C, and 0.57 °C, respectively. As a result, the temperature difference decreases as the field frequency increases in the first 5 min. In the second time period, however, the field frequency of 200 kHz accounts for the highest temperature increments (6.55 °C), while the lowest temperature value (5.83 °C) was measured at 400 kHz. The maximum temperature (30.68 °C) is recorded at the field frequency of 100 kHz; hence, the most heating was created over the 10 min of exposure time at 200 kHz. As the only variable in the aforementioned equation is ∆T/∆t, SAR is proportional to the rate of temperature change or the slope of the lines in Fig. S1a. Each of the examples may has two SAR values calculated due to the two time periods. Fig. S1b shows the SAR results for two different times as a function of the field frequency. As the field frequency rises, the SAR values in the first-time interval at 200 kHz, 300 kHz, and 400 kHz are 107.8 (W/g), 95.8 (W/g), and 105.2 (W/g) respectively. The SAR are 31.2, 21.6, 8.4, and 35.2 correspondingly for field frequencies of 100 kHz,200 kHz, 300 kHz, and 400 kHz at the second time period. The highest SAR that can be obtained in this experiment is 107.8 w/g at 200 kHz, which is higher than the one that occurs in the first five minutes at 300 kHz. For the first, second, and whole-time intervals, the mean values of SAR are computed as 84.55 (W/g), 24.1 (W/g), and 54.32 (W/g) respectively (see supplementary information file, Fig. S1a,b, Tables S1, S2).

## Conclusion

In connection with recent advancements in biomedical applicability of herbal seeds, in this relevant research study, the primary prospect was to design and synthesis an innovative magnetic nanobiocomposite fabricated by extracted flaxseed mucilage hydrogel, silk fibroin solution. Following the composition of these two natural-based components and the in-situ synthesis of Fe_3_O_4_ MNPs in the presence of natural composite substrate, magnetic flaxseed mucilage hydrogel/SF nanobiocomposite resulted with multifunctional biological activities. To describe more, in accordance with in-vitro cytotoxicity assay and examining two different normal HEK293T (95.73%, 96.19%) and breast cancer BT549 (87.32%, 86.9%) cell lines in 2 and 3 days, it could be deduced that despite using the highest concentration of magnetic flaxseed mucilage hydrogel/SF nanobiocomposite (1.75 mg/mL), the new magnetic nanostructure demonstrated high biocompatibility in the presence of normal HEK293T cell line and as well, it could prevent the growth of breast cancer BT549 cell line. Another privilege of this new magnetic nanobiocomposite was the low hemolytic effect (less than 5%) which disclosed its hemocompatibility with red blood cells. Furthermore, 1 mg/mL of magnetic flaxseed mucilage hydrogel/SF nanobiocomposite was sufficient to generate a high SAR value (107.8 w/g) at 200 kHz. Over all, as attested by in-vitro biological experiments, this new magnetic nanobiocomposite a can be considered as an appealing candidate for further biomedical applications like cancer treatment and tissue engineering.

## Materials and methods

### Materials

Aside from silkworm cocoons, all of the chemical reagents and chemical solvents including ethylenediaminetetraacetic acid (anhydrous, ≥ 99.0%), tris (hydroxymethyl) aminomethane (≥ 99.8%), anhydrous sodium carbonate (powder, 99.99%), lithium bromide (anhydrous, ≥ 99.0%), ammonia (≥ 99.98%), iron (III) chloride hexahydrate (reagent grade, ≥ 98%), and iron (II) chloride tetrahydrate (reagent grade, 98%) were purchased from Sigma-Aldrich company. In addition to this, the dialysis tubing cellulose membrane with a determined molecular weight cut-off (14 kDa) was prepared by Sigma-Aldrich company too.

### Plant materials

Brown Flax (*L. usitatissimum*) seeds used in this study was purchased from medicinal plants market in Tajrish, Tehran, Iran. This research study complies with local and national guidelines, and as well, International Union for Conservation of Nature (IUCN) policy statement.

### Extraction of flaxseed mucilage hydrogel

According to the reported extraction method of flaxseed mucilage hydrogel^57^, with some modifications, first, the cleaned seeds were submerged in distilled water to remove any additional materials. Afterwards, the flaxseeds were dried at 45 °C for 12 h. After the mentioned time and given a determined ratio (1:20 w/v), the dried flaxseeds were mixed with distilled water and kept in the stirring condition at 60 °C for 2 h. In the next step, the obtained mucilage hydrogel was filtered using a double layer cheesecloth to separate the seeds and then centrifuged at 10,000 rpm for 25 min to eliminate the remained impurities. Some part of purified flaxseed mucilage hydrogel was stored at 4 °C for the next synthesis steps. Besides, for further experiments, some part of it was poured into petri dishes to pre-prepare for the freeze-drying process and it was kept at − 70 °C for 24 h. Finally, the solvent sublimation was conducted by putting the frozen petri dishes into the freeze-dryer device with a determined temperature and pressure condition (− 60 °C, 0.1 bar) for 24 h.

### Extraction of silk fibroin

The extraction process of silk fibroin was performed according to the previous degumming methods^58,59^. First, small pieces of three cut silkworm cocoons were boiled in an aqueous Na_2_CO_3_ solution (0.21% w/v) for 2 h in order to remove and separate the glue-like sericin proteins from the cocoons structure. After 2 h, the elution of degummed silk fibers was conducted by distilled water several times and then, the clean fibers were dried at room temperature for 12 h. In the next step, the degummed silk fibers which were weighted before, were hydrolyzed using a determined volume of fresh LiBr Solution (9.3 M, in H_2_O). Following that, the clear solution obtained from hydrolysis process, was kept in the stirring condition at 60 °C for 2 h. After the mentioned time, to eliminate any impurities and excess amount of LiBr, the prepared clear solution was dialyzed using a dialysis tubing cellulose membrane in the presence of distilled water and as well, the dialysis process was continued for three days at room temperature. Eventually, the purified SF solution egressed from the dialysis tube, was kept at 4 °C for the next synthesis step.

### Preparation of flaxseed mucilage hydrogel/SF structure

As mentioned in the previous research studies^60,61^, by considering some modifications, 12 mL of prepared flaxseed mucilage hydrogel and 12 mL of fresh SF solution were mixed together and stirred for 12 h at room temperature condition. After 12 h, the mixture solution was poured into petri dishes to be pre-prepared for the freeze-drying process and then, they were kept at freezer (− 70 °C) for 24 h. After the relevant time, the solvent sublimation was carried out by putting the frozen petri dishes into the freeze-dryer device with a determined temperature and pressure condition (− 60 °C, 0.1 bar) for 24 h.

### Preparation of magnetic flaxseed mucilage hydrogel/SF nanobiocomposite

Following the Preparation of flaxseed mucilage hydrogel/SF structure, first, in a round flask bottom, 12 mL of prepared flaxseed mucilage hydrogel and 12 mL of fresh SF solution were mixed together and stirred for 12 h at room temperature condition. After 12 h, 5.82 g of FeCl_3_.6H_2_O and 2.66 g of FeCl_2_.4H_2_O were added to the mixture solution. Next, by providing the N_2_ atmosphere condition, the reaction mixture was stirred at 70 °C for 30 min. Following that, 25 mL of 25% aqueous ammonia was drop wisely added to the mixture solution in 30 min and then it was continuously stirred at 70 °C for 2 h. After the mentioned time (2 h) and cooling of the reaction solution, the black precipitate obtained from the reaction was separated using an external magnet and it was eluted with distilled water several times to remove the unreacted reagents and reach a neutral pH value (pH = 7). Finally, the prepared magnetic flaxseed mucilage/SF nanobiocomposite was dried at 60 °C for 24 h.

### *Preparation of bare Fe*_*3*_*O*_*4*_* MNPs*

The bare Fe_3_O_4_ MNPs was synthesized in accordance with reported co-precipitation method^62^. First, in a round flask bottom, 5.82 g of FeCl_3_.6H_2_O and 2.66 g of FeCl_2_.4H_2_O were dissolved in 24 mL of distilled water. By salts disbandment, the mixture solution was kept under the N_2_ atmosphere condition and heated up to 70 °C. After settling a constant temperature (70 °C), 25 mL of aqueous ammonia (25%) was drop wisely added to the mixture solution in 30 min. Next, the mixture solution was continuously stirred for 2 h. Following the mentioned time (2 h) and cooling the reaction solution, the black precipitate was separated using an external magnet, washed with distilled water several times to reach a neutral pH value (pH = 7) and eliminate any unreacted reagent. Subsequently, the prepared Fe_3_O_4_ MNPs was dried at 60 °C for 24 h.

### Fourier-transform infrared spectroscopy

To characterize the functional groups in each synthesis step, all the FT-IR spectra were taken using Fourier-transform infrared (FT-IR) spectrometer (Perkin Elmer,1720-X model, USA). The preparation of sample pellets was conducted by mixing 0.1 to 1.0% of each sample with 200 to 250 mg of fine KBr powder. Given the frequency range (400–4000 cm^−1^), a determined number of scan (32 times), and spectral resolution (2 cm^−1^), all the spectra were recorded at room temperature.

### Field-emission scanning microscopy and energy-dispersive X-ray spectroscopy

Operating at a 15 kV, the structure and morphology of all samples from each synthesis step were determined using the field-emission scanning microscope (FE-SEM) (ZEISS-Sigma VP model, Germany). Each sample was mounted on a double-side carbon tape and subsequently, stainless-steel stub. Afterwards, each sample was sputter-coated with gold (Agar Sputter Coater model, Agar Scientific, England). Characterizing the elemental structure of magnetic flaxseed mucilage hydrogel/SF nanobiocomposite was performed by EDS detector (Oxford instrument, England), which was attached to the mentioned field-electron scanning microscope. Additionally, the elemental mapping images were recorded to determine the distribution pattern of structural elements of magnetic flaxseed mucilage hydrogel/SF nanobiocomposite^60^.

### Transmission electron microscopy

Apart from the morphology, structure, the distribution of Fe_3_O_4_ MNPs in the structure of magnetic flaxseed mucilage hydrogel/SF nanobiocomposite was conducted by transmission electron microscope (TEM) (Philips, EM 208S model, Netherlands) with 200 Kx magnification.

### X-ray diffraction pattern

X-ray diffraction (XRD) pattern of magnetic flaxseed mucilage hydrogel/SF nanobiocomposite was recorded using Brucker X-ray diffractometer device (D8 Advanced Model, USA). The device was equipped with Lynxeye detector (0D mode), and Cu-Kα radiation (λ = 0.154 nm, 40 kV, 40 mA). The angle scan was in the range of 5° ≤ 2*θ* ≤ 90° with 0.2°/s scan rate^60^.

### Vibrating-sample magnetometer

Vibrating-sample magnetometer (VSM) (LBKFB model magnetic kavir, Iran) was used to evaluate and compare the magnetic property of bare Fe_3_O_4_ NMPs and magnetic flaxseed mucilage hydrogel/SF nanobiocomposite. All the hysteresis loop curves were recorded by an applied magnetic field from − 10,000 to + 10,000 Oe.

### Thermogravimetric analysis

Thermogravimetric (TG) behavior of bare Fe_3_O_4_ MNPs, freezed dried forms of flaxseed mucilage hydrogel, silk fibroin solution, and as well, magnetic flaxseed mucilage hydrogel/SF nanobiocomposite was analyzed by thermogravimetric analyzer instrument (TGA2 model, Mettler Toledo, USA). Given the nitrogen atmosphere, and using an alumina pan, 5.0 mg of designed magnetic nanobiocomposite was applied for this analysis. Besides, the thermal cycle was run between room temperature (25 °C) to 600 °C with a constant heating rate (10 °C/min).

### In-vitro cytotoxicity assay

The toxicity of bare Fe_3_O_4_ MNPs and the magnetic flaxseed mucilage hydrogel/SF nanobiocomposite were determined using MTT assay, according to our previous studies. First, the BT549 (breast cancer) cell line and HEK293T (human embryonic kidney) cell line were provided from the Pasteur Institute of Iran. Next, the proper culture medium (DMEM/F12, 10% fetal bovine serum (FBS), and 1% pen-strep) was prepared and the cells were cultured at 5 × 10^3^ cell/well in 96 well plate. Then, serially dilutions of designed magnetic nanobiocomposite with concentrations of 0.015, 0.031, 0.062, 0.125, 0.25, 0.5, 0.75, 1, 1.25, 1.5 and 1.75 mg/mL were added to each well and incubated for 48 h and 72 h. Cisplatin and the culture medium without any additive were used as the positive and negative controls, respectively. The cells were then treated with 3-(4,5 dimethylthiazol-2-yl)-2,5-diphenyl tetrazolium bromide (MTT) solution and incubated for 4 h at 37 °C. Next, 1% SDS was added to the wells and incubated for 16 h at 37 °C. Finally, the optical density of samples was measured at 550 nm using a microplate reader spectrophotometer (BioTeK, USA). All tests were repeated three times^60,63^. The percentage of toxicity and cell viability were calculated by Eqs. (2, 3).

2 $$\mathrm{Toxicity }\left(\mathrm{\%}\right)=\left( 1-\frac{\mathrm{mean \; OD \; of \;sample}}{\mathrm{mean\; OD \;of \; control}}\right) \times 100$$

3 $${\text{Cell viability }}\left( \% \right) \, = { 1}00 \, \left( \% \right) \, {-}{\text{ Toxicity}}\left( \% \right)$$

### In-vitro hemocompatibility assay

Hemolysis assay was performed based on the method presented in our previous studies to measure the blood compatibility of bare Fe_3_O_4_ MNPs and the magnetic flaxseed mucilage hydrogel/SF nanobiocomposite on human red blood cells (RBCs). A volunteer with O blood type was first selected and after completing informed consent form, fresh blood samples were taken from him. In the next step, RBCs were washed and diluted with NaCl 0.9% in a ratio of 2:100. Next, 100 µL of washed RBCs was poured onto a 96-well V-shaped bottom plate (Citotest, China). Then, each well received 100 µL of dispersed magnetic nanobiocomposite in NaCl 0.9% with concentrations of 0.25, 0.5, 0.75, 1 and 2 mg/mL. Triton X-100 was also used as positive control. After 2 h of incubation at 37 °C, the plate was centrifuged at 2000 rpm for 10 min. Finally, the supernatant of each well was transferred to the flat bottom plate and the OD was measured at 405 nm by the ELISA reader (Biohit, Finland)^64,65^. The hemolysis percentage was calculated by following Eq. (4) ^66^.

4 $$\mathrm{Hemolysis} \left(\%\right)=\left(\frac{\mathrm{mean \; OD \; of \; sample}-\mathrm{mean \; OD \; of \; negative \; sample}}{\mathrm{mean \; OD \; of \;positive \;control}-\mathrm{mean \;OD\; of \;negative \;control}}\right)\times 100$$

### Ethical issues

This study was performed in accordance with the principles outlined in the Declaration of Helsinki. Also, the experimental methods and the procedure for taking informed satisfaction were approved by Ethics Research Committee of Pasteur Institute of Iran.

### Statistical analysis

Statistical analysis for the comparison all biocompatibility and hemocompatibility results was accomplished by a t-test by SPSS Statistics 22.0 software (SPSS Inc. Chicago, IL, USA). The values of P ≥ 0.05 (*), P ≤ 0.05 (**) and P ≤ 0.001 (***) were considered as statistically insignificant, significant and very significant, respectively.

### Hyperthermia experiments

To assess the hyperthermia capacity of the magnetic flaxseed mucilage hydrogel/SF nanobiocomposite as a new candidate for magnetic fluid hyperthermia treatment, a determined amount of designed magnetic nanobiocomposite (1 mg/mL) was analyzed with a hyperthermia device (NATSYCO, Iran) considering a defined field amplitude (1000 W) and different frequencies (100 kHz, 200 kHz, 300 kHz, and 400 kHz).

### Supplementary Information

Supplementary Information.

## Data Availability

The datasets used and/or analysed during the current study available from the corresponding author on reasonable request.
