# Supplementary material for: Design and fabrication of a magnetic nanobiocomposite based on flaxseed mucilage hydrogel and silk fibroin for biomedical and in-vitro hyperthermia applications
Source: Sci Rep. 2023 Nov 27;13:20845. doi: 10.1038/s41598-023-46445-w (PMC10681992; doi:10.1038/s41598-023-46445-w)
Supplement: Supplementary file 1 — Supplementary Information. [file 41598_2023_46445_MOESM1_ESM.docx]

**Supplementary information**

**Design and fabrication of a magnetic nanobiocomposite based on Flaxseed mucilage hydrogel and silk fibroin for biomedical and in-vitro hyperthermia applications**

Fateme Radinekiyan^a,b^, Reza Eivazzadeh-Keihan^b^, Mohammad Reza Naimi-Jamal^a^*, Hooman Aghamirza Moghim Aliabadi^c^, Milad Salimi Bani^d^, Shirin Shojaei^e^, Ali Maleki ^b^*

*^a^Research Laboratory of Green Organic Synthesis and Polymers, Department of Chemistry, Iran University of Science and Technology, P.O. Box 16846‑13114, Tehran, Iran. Email:* [*naimi@iust.ac.ir*](mailto:naimi@iust.ac.ir)

*^b^Catalysts and Organic synthesis Research Laboratory, Department of Chemistry, Iran university of Science and Technology, Tehran 16846-13114, Iran. Email:* [*maleki@iust.ac.ir*](mailto:maleki@iust.ac.ir)*; Fax: +98-21-73021584; Tel: +98-21-73228313*

*^c^Advanced Chemical Studies Lab, Department of Chemistry, K. N. Toosi University of Technology, Tehran, Iran.*

*^d^Department of Biomedical Engineering, Faculty of Engineering, University of Isfahan, Isfahan, Iran*

*^e^Medical School of Pharmacy, Nanotechnology Department, Kermanshah University of Medical Science, Kermanshah, Iran*

*Corresponding authors: (M. R. Naimi-Jamal, A. Maleki)

| Table of contents | | |
| --- | --- | --- |
| Entry | **Subject** | **Page** |
| 1 | Fig. S1. a) Relevance between time and temperature in different frequencies by using a determined amount of magnetic flaxseed hydrogel/SF nanobiocomposite (1 mg/mL), and b) relevance between SAR and field frequency in different time intervals using a determined amount of magnetic flaxseed hydrogel/SF nanobiocomposite (1 mg/mL). | S3 |
| 2 | Table S1. Temperature rise at different frequencies. | S4 |
| 3 | Table S2. SAR values for all the frequencies | S4 |

**
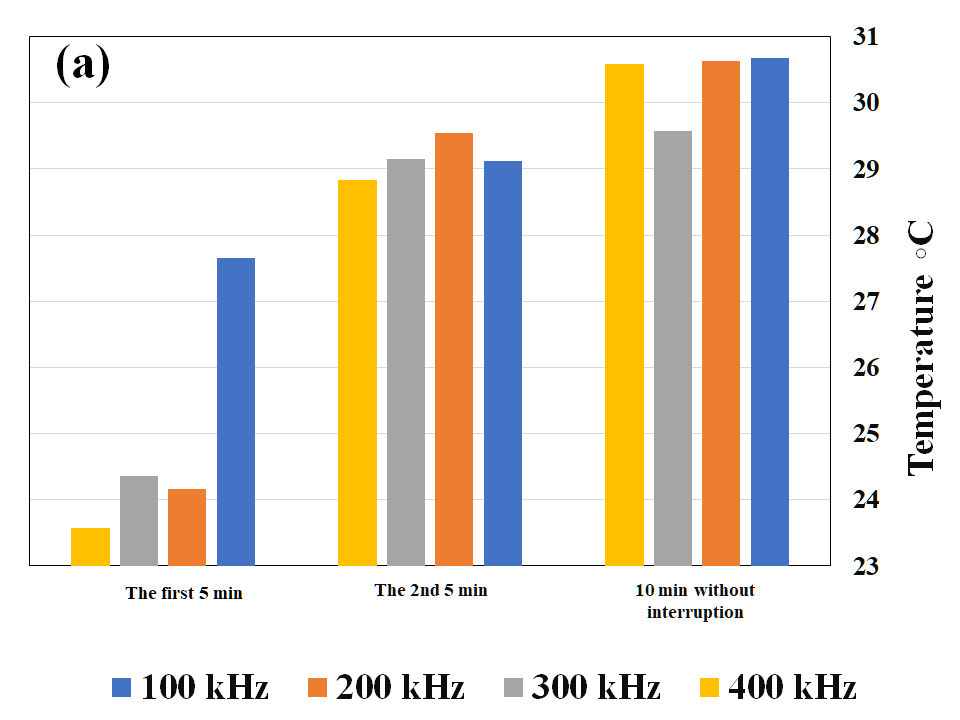
**

**
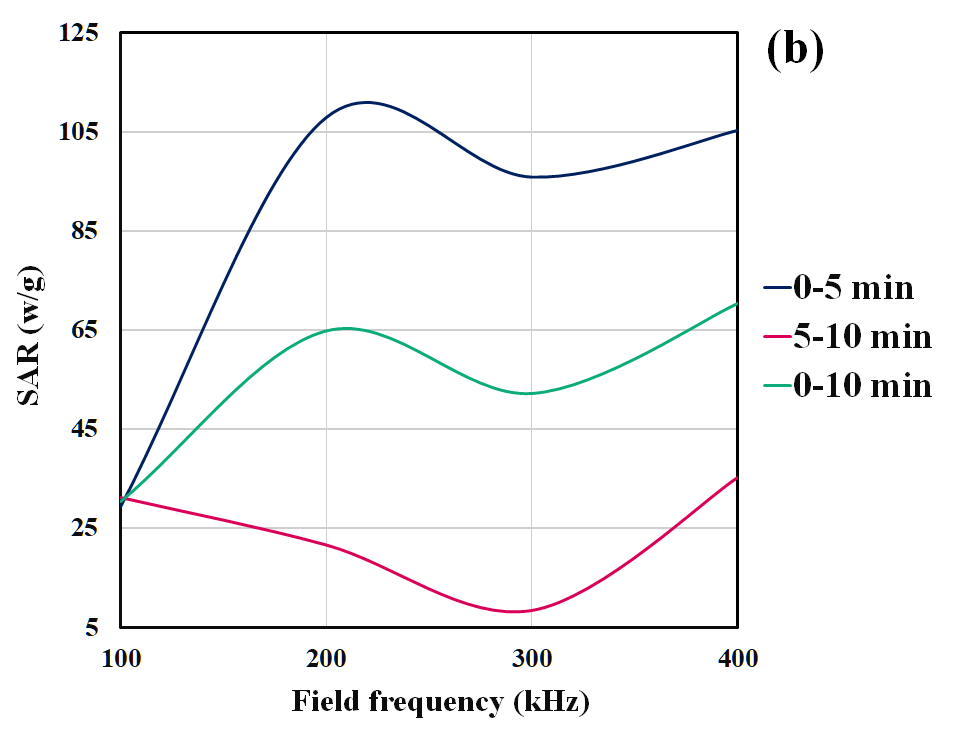
**

**Fig. S1.** a) Relevance between time and temperature in different frequencies by using a determined amount of magnetic flaxseed hydrogel/SF nanobiocomposite (1 mg/mL), and b) relevance between SAR and field frequency in different time intervals using a determined amount of magnetic flaxseed hydrogel/SF nanobiocomposite (1 mg/mL).

**Table S1.** Temperature rise at different frequencies.

| Time (min) | f = 100 kHz | f = 200 kHz | f = 300 kHz | f = 400 kHz |
| --- | --- | --- | --- | --- |
| The first 5 min | 27.65 | 24.16 | 24.36 | 23.57 |
| The 2nd 5 min | 29.12 | 29.55 | 29.15 | 28.83 |
| 10 min without  interruption | 30.68 | 30.63 | 29.57 | 30.59 |

**Table S2.** SAR values for all the frequencies

| SAR (W/g) | Δt = 0-5 min | Δt = 5-10 min | Δt = 0-10 min |
| --- | --- | --- | --- |
| f = 100 kHz | 29.4 | 31.2 | 30.3 |
| f = 200 kHz | 107.8 | 21.6 | 64.7 |
| f = 300 kHz | 95.8 | 8.4 | 52.1 |
| f = 400 kHz | 105.2 | 35.2 | 70.2 |
